# Case Report: Rapid progression of inflammation-driven coronary artery lesions in a normolipidemic patient with ANCA-associated vasculitis complicated by Stanford type A aortic dissection

**DOI:** 10.3389/fimmu.2026.1736895

**Published:** 2026-03-12

**Authors:** Guangzhen Lu, Xiaoting Wang, Xinye Wang, Hong Zhuang, Gang Zhao

**Affiliations:** 1 Department of Cardiology, Shandong Provincial Hospital Affiliated with Shandong First Medical University, Jinan, Shandong, China; 2 Department of Otorhinolaryngology, Shandong Provincial Hospital Affiliated with Shandong First Medical University, Jinan, Shandong, China

**Keywords:** ANCA-associated vasculitis, case report, inflammatory atherosclerosis, IVUS, rapid coronary artery disease progression, Stanford type A aortic dissection

## Abstract

**Background:**

Antineutrophil cytoplasmic antibody (ANCA)-associated vasculitis (AAV) is a systemic autoimmune small- to medium-vessel vasculitis. Reports of rapidly progressive left main/left anterior descending (LM/LAD) disease under normolipidemic conditions with parallel coronary–aortic progression are scarce.

**Case presentation:**

We report a case of perinuclear antineutrophil cytoplasmic antibody (p-ANCA) and myeloperoxidase (MPO)–positive ANCA-associated vasculitis (AAV) in which, despite persistently normal lipid levels, the coronary arteries developed rapidly progressive stenosis and occlusion within one year. Intravascular ultrasound (IVUS) revealed concentric thickening of the coronary arterial wall characterized predominantly by soft plaques with minimal calcification, which was consistent with an inflammation-driven remodeling phenotype. Because the admission electrocardiogram (ECG) revealed an ST-segment elevation myocardial infarction (STEMI)-equivalent pattern (aVR ST elevation with diffuse ST depression), the patient was treated for acute myocardial infarction, and urgent LM-to-LAD drug-eluting stent (DES) implantation was performed. During a four-month follow-up after discharge, no recurrent angina or ischemic events were reported.

**Conclusion:**

In a p-ANCA/MPO-positive AAV patient with sustained normolipidemia, we observed rapidly progressive LM/LAD disease occurring in parallel with aortic involvement, which was consistent with an inflammation-driven coronary phenotype that precipitated acute coronary syndrome. Integrating sequential coronary–aortic imaging with inflammatory biomarker surveillance and individualized immunomodulatory and antithrombotic strategies may enable earlier risk detection and optimize outcomes in patients with AAV.

## Introduction

Antineutrophil cytoplasmic antibody (ANCA)-associated vasculitis (AAV) is an immune-mediated disorder that primarily affects small- to medium-sized vessels, and cardiovascular involvement is closely associated with poor prognosis (1). Previous studies have indicated that inflammation-driven vascular remodeling can precipitate coronary artery events even when lipid levels remain within the normal range (2, 3). However, progression from coronary artery disease-reporting and data system (CAD-RADS) 1 to severe left main and left anterior descending (LAD) stenosis accompanied by Stanford type A aortic dissection within approximately one year is exceedingly rare.

Here, we report a patient with perinuclear antineutrophil cytoplasmic antibody (p-ANCA)/myeloperoxidase (MPO)-positive AAV who developed acute myocardial infarction with an ST-segment elevation myocardial infarction (STEMI)-equivalent electrocardiogram (ECG) pattern—ST elevation in aVR with diffuse ST-segment depression—suggestive of left main or proximal multivessel ischemia during a phase of heightened systemic inflammation. Intravascular ultrasound (IVUS) revealed a characteristic inflammation-dominant coronary phenotype. This case study aims to supplement existing evidence and explore the potential underlying pathophysiological mechanisms and clinical management considerations.

## Case description

### Initial diagnosis and early course (February 2024 – April 2024)

The patient’s clinical trajectory began in February 2024, when the 56-year-old female presented to an outside hospital with recurrent cough and sputum production. At that time, chest computed tomography (CT) revealed thickening of the ascending aortic wall with an intimal-like shadow, narrowing of the right pulmonary artery trunk, and multiple inflammatory pulmonary lesions. Subsequent pulmonary artery and abdominal aortic CT angiography (CTA) demonstrated signs of pulmonary hypertension and filling defects in the right lower lobe pulmonary artery branches, suggestive of embolism. Notably, a dedicated coronary CTA performed during this initial evaluation revealed no significant coronary abnormalities.

Laboratory testing confirmed positivity for antineutrophil cytoplasmic antibodies (ANCAs), specifically myeloperoxidase (MPO)-ANCA and proteinase 3 (PR3)-ANCA, establishing a diagnosis of ANCA-associated vasculitis (AAV). The clinical phenotype—characterized by p-ANCA/MPO positivity, the absence of asthma or eosinophilia, and a lack of granulomatous ENT manifestations—favored microscopic polyangiitis (MPA) over granulomatosis with polyangiitis (GPA) or eosinophilic granulomatosis with polyangiitis (EGPA). Although chest CT showed an interstitial lung disease (ILD) pattern (**Supplementary Figure 1**) described radiologically as “connective tissue disease (CTD)-related,” this was interpreted as consistent with AAV-associated pulmonary involvement in accordance with the 2022 ACR/EULAR classification criteria for MPA (4). Immunosuppressive therapy was initiated with prednisone acetate (starting dose unavailable), alongside rosuvastatin and anticoagulation; however, the patient self-discontinued these medications after approximately one month.

### Systemic progression and aortic involvement (April 2024 – November 2024)

**Figure 1:**
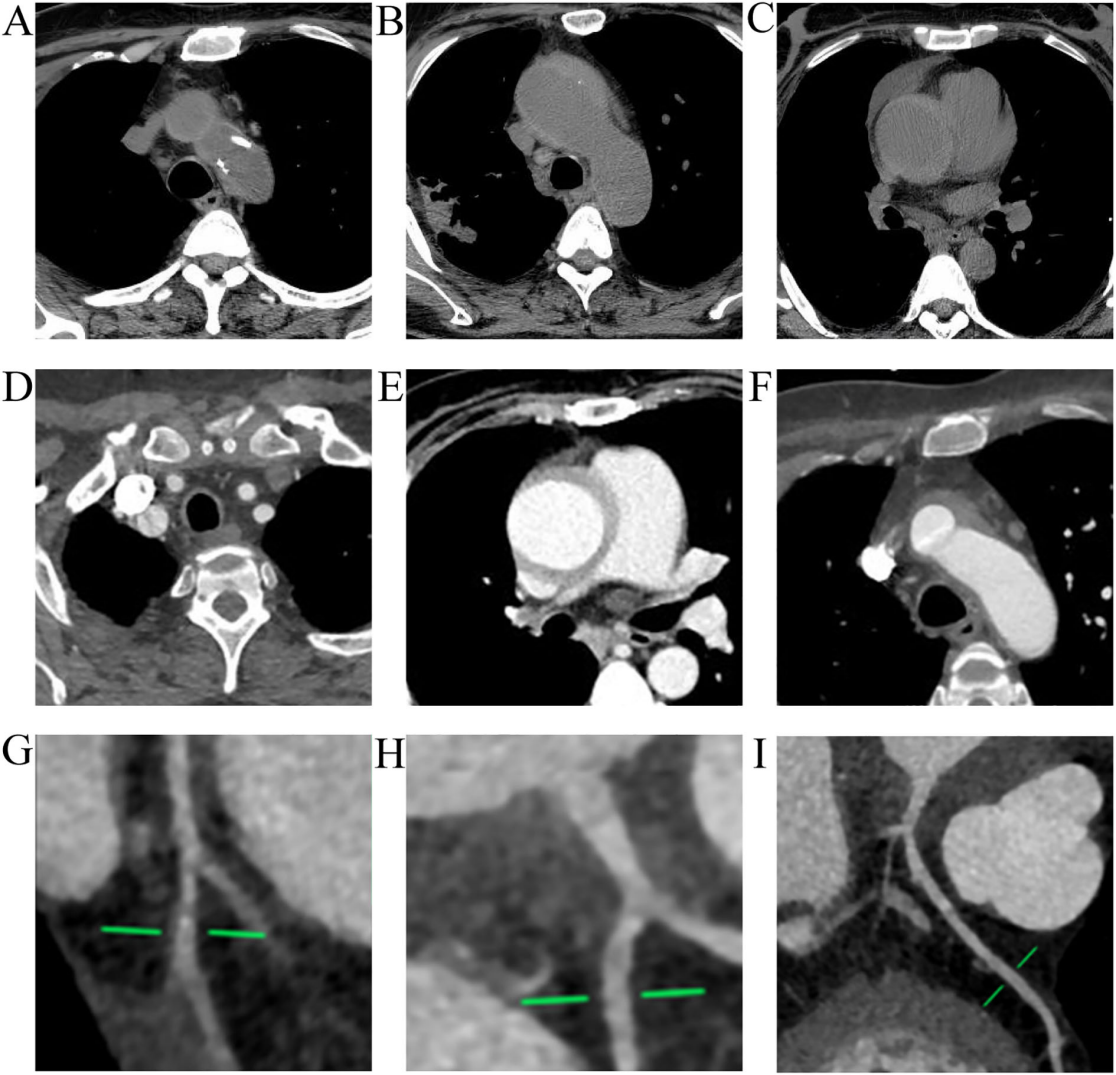
Whole-aorta CTA and coronary CTA prior to the index acute coronary event. **(A–F)** Whole-aorta computed tomography angiography (CTA) revealed Stanford type A aortic pathology, with mural abnormalities consistent with an intramural hematoma and/or dissection involving the ascending aorta and aortic arch, with extension to the brachiocephalic (innominate) artery origin. **(D)** shows the brachiocephalic artery origin; panels **(A, B, F)** show the aortic arch; and **(C, E)** show the ascending aorta. **(G–I)** Coronary CTA obtained during the same evaluation. **(G, H)** depict the left circumflex artery (LCx) and its branches, and **(I)** depicts the left main (LM) and left anterior descending (LAD) arteries. Although the initial CTA report did not identify focal obstructive stenosis, a retrospective review demonstrated subtle diffuse tapering of the LM (green markers), which later correlated with severe LM involvement on invasive coronary angiography during NSTEMI admission. ACS, acute coronary syndrome; CTA, computed tomography angiography; IMH, intramural hematoma; LAD, left anterior descending artery; LCx, left circumflex artery; LM, left main coronary artery; NSTEMI, non–ST-segment elevation myocardial infarction; RCA, right coronary artery.

Following the cessation of therapy, the patient developed recurrent high fevers (peaking at 39.4 °C) in April 2024, accompanied by ocular pain, visual decline, and hearing loss. She presented to our outpatient clinic in May 2024, where a non-contrast chest CT revealed findings suspicious for ascending aortic dissection (**Figures 1A–C**). Although immediate cardiovascular surgery consultation was advised, the patient did not attend. Immunosuppressive therapy was re-initiated with prednisone (10 mg twice daily) and mycophenolate mofetil (MMF, 0.5 g twice daily).

In July 2024, the patient was admitted to our Rheumatology Department with persistent disease activity (MPO antibody 61.20 CU; p-ANCA 1:10). Repeat chest CT confirmed an ascending aortic dissecting aneurysm with a small pericardial effusion. Concurrently, comparison with the prior scan (May 2024) revealed radiological progression of the interstitial lung disease, particularly within the right lung (**Supplementary Figure 1**). To control the systemic inflammation, prednisone was escalated to 15 mg three times daily, and intermittent intravenous cyclophosphamide (IVCY) pulses were initiated (cumulative dose 2.8 g from July 2024 to May 2025).

A follow-up whole aorta CTA performed on November 22, 2024, revealed an intramural hematoma (Stanford type A) involving the ascending aorta and extending to the brachiocephalic artery origin (**Figures 1D–F**). Cardiothoracic surgery recommended close medical management and dynamic surveillance rather than immediate repair, given the thinness of the hematoma (<1 cm) and the absence of pericardial effusion (5). Concurrent coronary CTA performed on the same day demonstrated mild atherosclerotic plaques (CAD-RADS 1) in the left main (LM) and left anterior descending (LAD) arteries Concurrent coronary CTA performed on the same day demonstrated mild atherosclerotic plaques (CAD-RADS 1) in the left main (LM) and left anterior descending (LAD) arteries. However, a retrospective review of this imaging later identified subtle, diffuse tapering of the left main coronary artery—a pattern that, while not meeting obstruction thresholds at the time, correlated with the subsequent progression to severe stenosis.

### Cardiovascular risk profile

Longitudinal profiling over the preceding year revealed a complex risk landscape. The patient’s cardiovascular risk profile was notable for a non-obese body habitus (BMI ~21.4–23.1 kg/m²), preserved renal function, normotension, and normolipidemia (LDL-C 2.38–2.73 mmol/L), with no history of smoking or premature coronary artery disease. However, glycemic control was markedly poor, with HbA1c levels fluctuating between 11.1% and 11.7%. While such metabolic derangement is a recognized driver of atherosclerosis, the clinical picture observed here—specifically the rapid progression within one year and the IVUS findings of concentric wall thickening with predominantly soft, minimally calcified plaques—diverges significantly from the heavily calcified, diffuse atherosclerosis typically associated with long-standing diabetes. These morphological features, evolving in parallel with active systemic vasculitis, strongly suggest an inflammation-dominant remodeling process rather than classic metabolic atherosclerosis.

### Acute coronary syndrome and intervention (June 2025)

**Figure 2:**
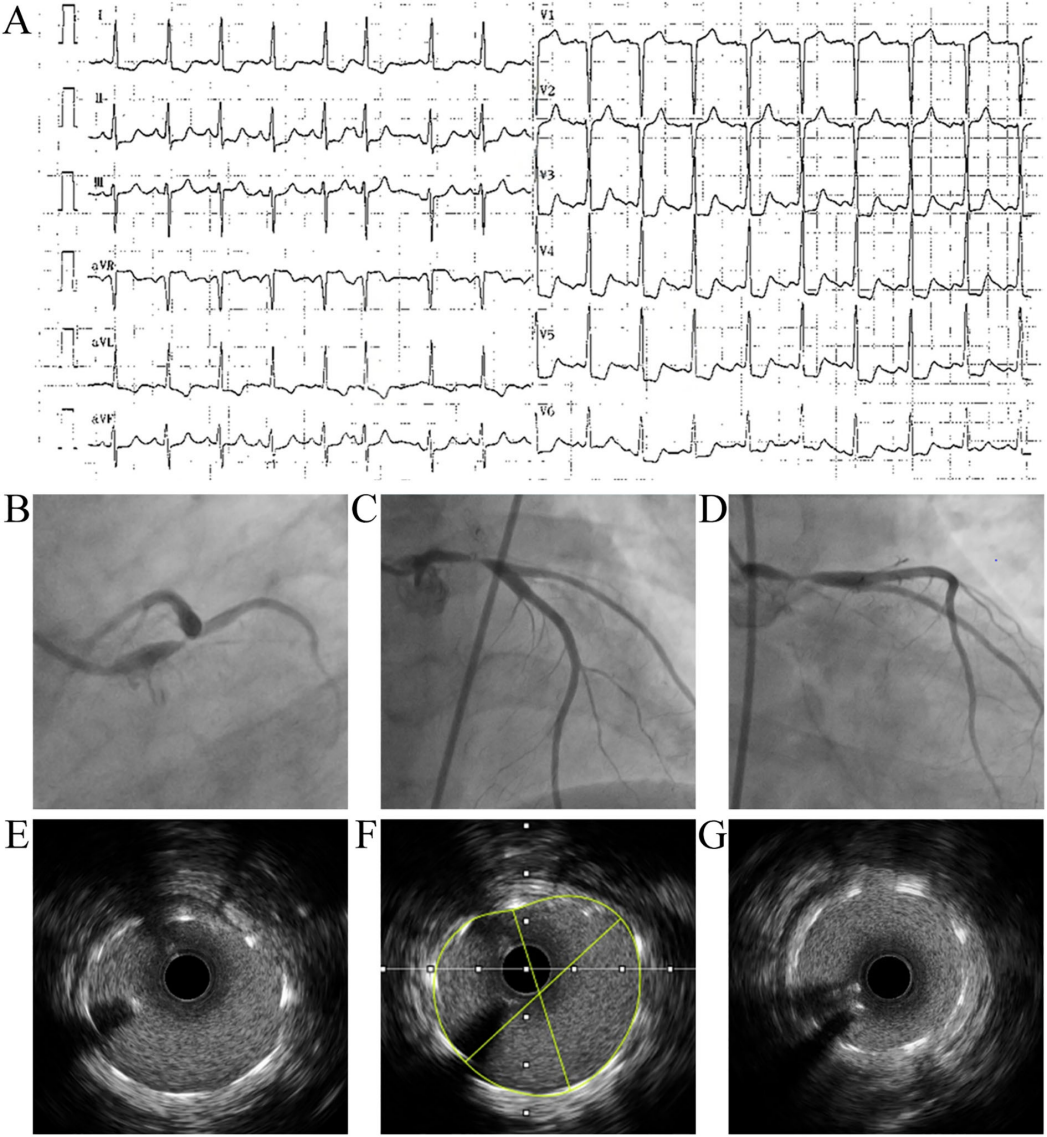
Electrocardiography, invasive coronary angiography, and intravascular ultrasound (IVUS) findings during the index admission. **(A)** Twelve-lead electrocardiogram at presentation showing ST-segment elevation in lead aVR with widespread ST-segment depression, which is consistent with a STEMI-equivalent ischemic pattern. **(B–D)** Invasive coronary angiography demonstrating severe left coronary involvement: **(B, C)** show critical disease involving the left main (LM) and left anterior descending (LAD) arteries, and **(D)** shows left circumflex (LCx) artery involvement. **(E–G)** Representative intraprocedural IVUS images acquired during PCI, demonstrating concentric vessel wall thickening, predominantly low-echo (“soft”) plaque, and minimal calcification; **(F)** shows the lumen contour tracing used for quantitative assessment. IVUS, intravascular ultrasound; LAD, left anterior descending artery; LCx, left circumflex artery; LM, left main coronary artery; PCI, percutaneous coronary intervention; STEMI, ST-segment elevation myocardial infarction.

**Figure 3:**
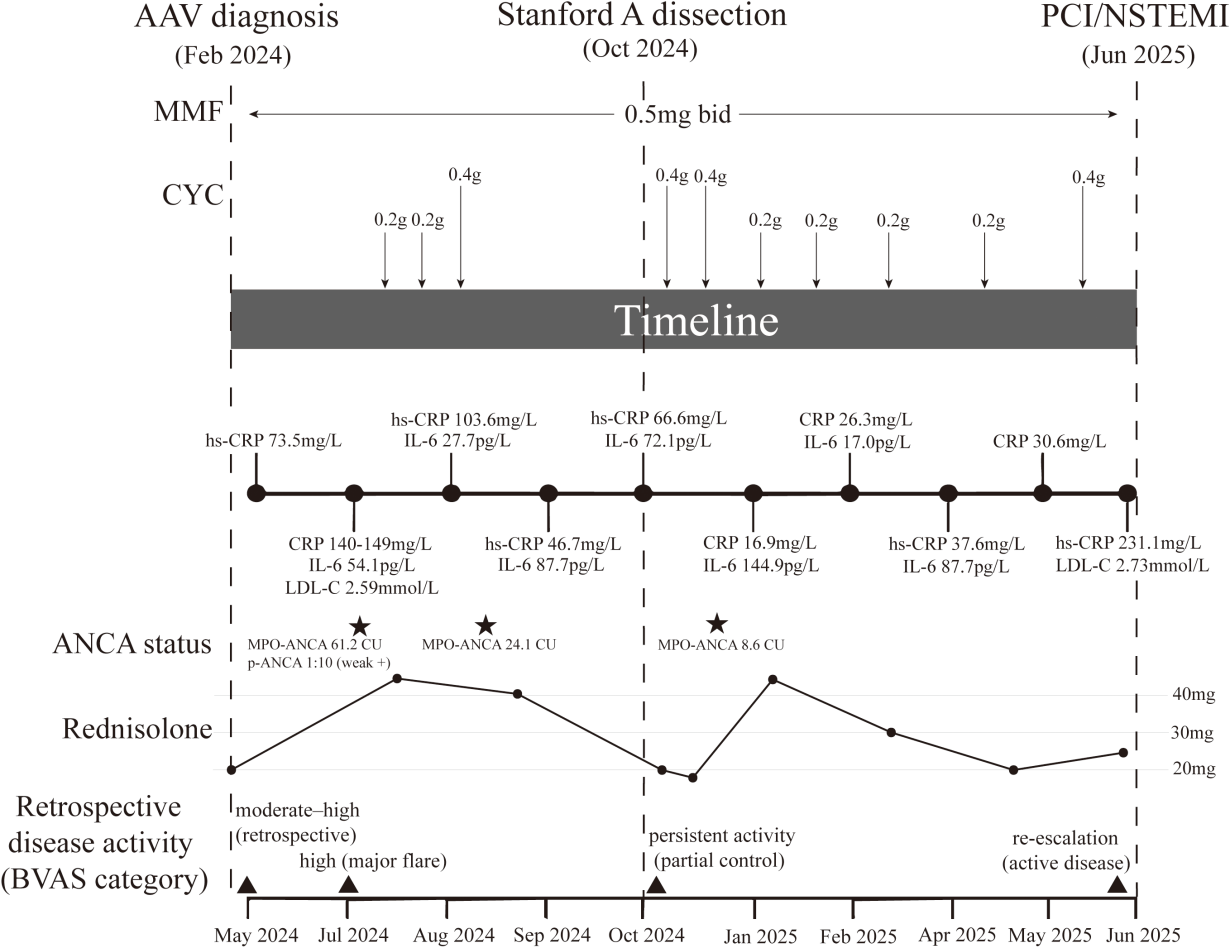
Clinical timeline linking immunosuppressive therapy, inflammatory activity, and cardiovascular events in MPO-ANCA–positive AAVs. The schematic summarizes the longitudinal course of disease activity and treatment from May 2024 to June 2025. Key clinical milestones are marked by vertical dashed lines, including AAV diagnosis (Feb 2024), Stanford type A aortic dissection/intramural hematoma (Oct 2024), and NSTEMI requiring PCI (Jun 2025). The top tracks depict immunosuppressive therapy: mycophenolate mofetil (MMF) maintenance (0.5 g twice daily) and intermittent intravenous cyclophosphamide (CYC) pulses (individual pulse doses annotated above arrows; exact dates and dosing details are provided in **Supplementary Table 1**). The prednisolone trajectory is shown as a line plot (dose in mg/day) over time. Serial inflammatory biomarkers (CRP/hs-CRP and IL-6) are plotted along the timeline (values shown at each sampling time point), and selected lipid values (e.g., LDL-C) are annotated when available. ANCA status is displayed as MPO-ANCA levels (CU) and p-ANCA titers where measured. The bottom panel provides a retrospective categorization of disease activity (BVAS category) at key time points, included for visualization of relative activity rather than as prospectively scored BVAS. The exact sampling dates and all the laboratory results are listed in the **Supplementary Tables**. AAV, ANCA-associated vasculitis; ANCA, antineutrophil cytoplasmic antibody; BVAS, Birmingham Vasculitis Activity Score; CRP, C-reactive protein; hs-CRP, high-sensitivity C-reactive protein; CYC, cyclophosphamide; IL-6, interleukin-6; LDL-C, low-density lipoprotein cholesterol; MMF, mycophenolate mofetil; NSTEMI, non–ST-segment elevation myocardial infarction; PCI, percutaneous coronary intervention.

Notably, the patient remained under close rheumatological follow-up. Just prior to the coronary event, on May 26, 2025, she received an intravenous cyclophosphamide pulse (0.4 g; cumulative dose ~3.0 g) and was maintained on prednisone (12.5 mg twice daily) and MMF (0.5 g twice daily). Despite this ongoing immunosuppressive regimen and serological remission (MPO-ANCA declined from 61.2 CU to 8.6 CU), the coronary anatomical progression continued. In June 2025, 16 months after the initial AAV diagnosis, the patient was admitted with recurrent retrosternal chest pain and dyspnea. Electrocardiography revealed a STEMI-equivalent pattern characterized by diffuse ST-segment depression and reciprocal ST elevation in lead aVR (**Figure 2A**). Biochemical evidence of acute myocardial injury was substantial, with markedly elevated high-sensitivity cardiac troponin T (hs-cTnT 1,143 pg/mL) and N-terminal pro-B-type natriuretic peptide (NT-proBNP 4,169 pg/mL), although creatine kinase (CK) remained within normal limits (49 U/L). Concomitantly, the patient exhibited a pronounced systemic inflammatory response: laboratory workup showed absolute neutrophilia (7.66×10^9^/L; reference: 1.8–6.3×10^9^/L) despite a normal total white blood cell count (8.86×10^9^/L), alongside a critically elevated high-sensitivity C-reactive protein (hs-CRP 231.08 mg/L). Due to the emergent need for revascularization, ANCA titers were not reassessed during this admission; however, the most recent evaluation in December 2024 had indicated serological remission (MPO-ANCA 8.6 CU). The overall longitudinal course of immunosuppressive therapy, inflammatory activity, and cardiovascular events is summarized in **Figure 3**.

Given the acute ischemic presentation, urgent invasive coronary angiography was performed, revealing critical progression compared to the CTA from seven months prior: 70–90% stenosis of the distal LM, 80–90% stenosis of the ostial LAD, and complete occlusion of the proximal left circumflex (LCx) artery (**Figures 2B–D**). Intravascular ultrasound (IVUS) demonstrated concentric arterial wall thickening characterized predominantly by soft plaques with minimal calcification (**Figures 2E, F**). This phenotype was consistent with inflammation-driven vascular remodeling rather than heavily calcified, diabetes-associated atherosclerosis.

**Figure 4:**
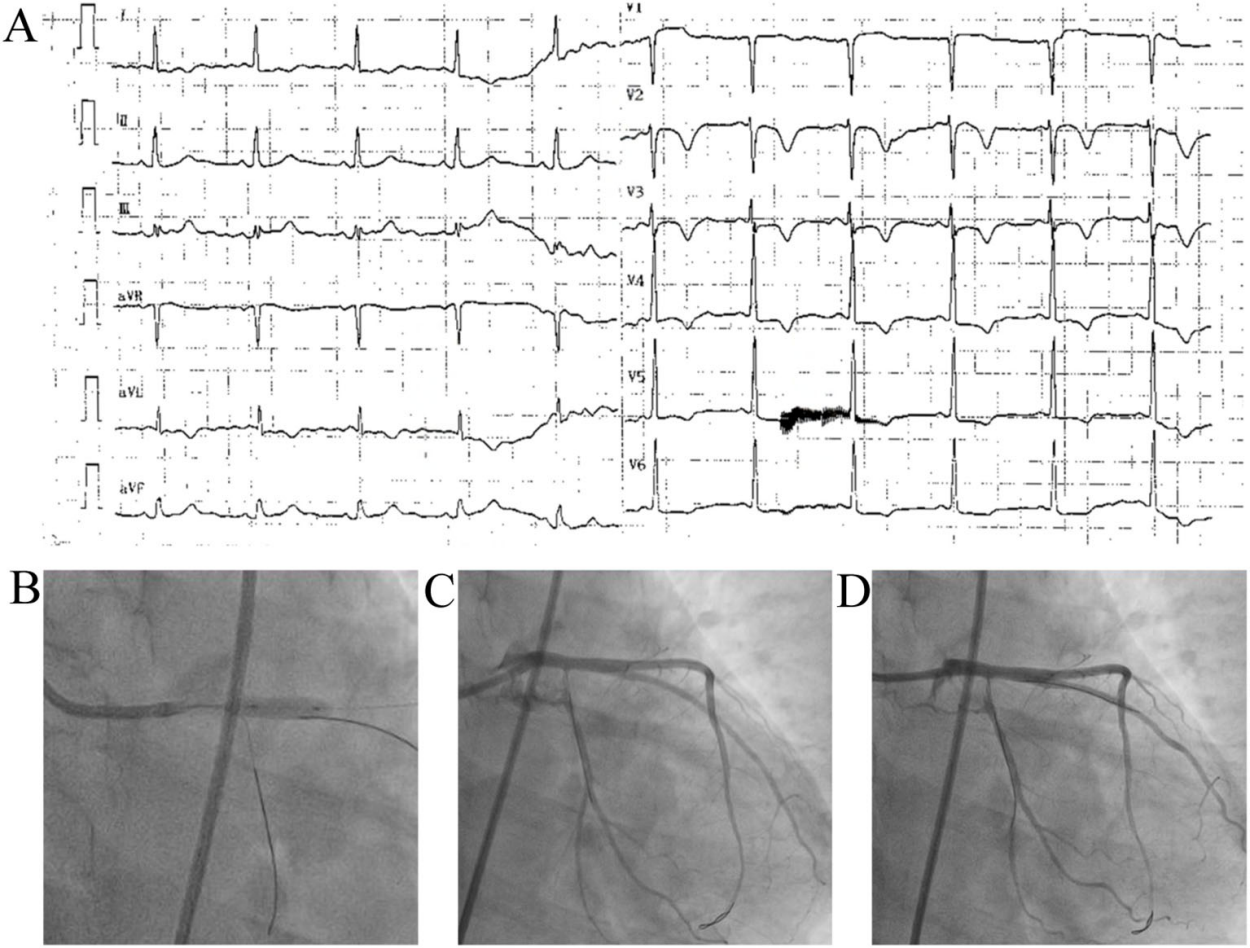
Electrocardiography and intraprocedural coronary angiography during PCI for the index NSTEMI. **(A)** Post–percutaneous coronary intervention (PCI) electrocardiogram showing persistent ST-segment depression and T-wave inversion in leads I, II, aVL, and V_3_-V_6_, which is consistent with residual anterior wall ischemic changes. **(B–D)** Coronary angiography following drug-eluting stent implantation from the left main (LM) artery to the left anterior descending (LAD) artery.

A 4.0 × 15 mm drug-eluting stent was successfully implanted from the LM to the LAD. The electrocardiogram obtained after PCI and the procedural coronary angiography are shown in **Figure 4**. Post-procedure IVUS confirmed optimal expansion with a minimal lumen area of 14 mm² and no complications. The patient was discharged on dual antiplatelet therapy, statins, insulin, and maintenance immunosuppression (prednisone 12.5 mg twice daily and MMF 0.5 g twice daily). At the 4-month follow-up, she remained angina-free with improved activity tolerance.

## Discussion

Building on the clinical timeline and multimodality imaging of our patient, we hypothesize that an MPO-ANCA-associated inflammatory cascade may have contributed to the rapid progression of coronary lesions under normolipidemic conditions—specifically, rapidly progressive LM/LAD disease—beyond what would be expected from traditional lipid-related pathways alone (6, 7). Notably, this coronary trajectory evolved in parallel with aortic involvement (Stanford type A dissection/intramural hematoma) within the AAV disease course, highlighting concomitant coronary-aortic progression in AAV. Mechanistically, the activation of neutrophils by MPO-ANCA triggers the excessive release of neutrophil extracellular traps, which in turn cause oxidative injury and the apoptosis of endothelial cells while exposing proteolytic enzymes such as myeloperoxidase and elastase, which degrade the vascular extracellular matrix (6, 8, 9). As a result, persistent endothelial dysfunction increases vascular permeability and promotes the continuous recruitment of leukocytes, thereby maintaining a chronic inflammatory environment within the coronary wall (7, 9). In line with this mechanism, our patient exhibited sustained elevations in systemic inflammatory markers, including IL-6, CRP, and the erythrocyte sedimentation rate (ESR), throughout key clinical stages, which supports the presence of ongoing innate immune activation rather than a short-lived infection-related response (7).

While coronary involvement in AAV has been described in isolation, this case distinguishes itself by demonstrating a fulminant, ‘pan-vascular’ progression where both large vessels (aorta) and medium vessels (coronary arteries) deteriorated simultaneously within a remarkably short timeframe (<16 months). Unlike previously published cases that often describe discrete coronary arteritis or slow-progressing atherosclerosis, our patient exhibited a parallel, rapid remodeling of the entire arterial tree under normolipidemic conditions. This highlights a distinct clinical phenotype: rapidly progressive, inflammation-driven multi-vessel failure that can precipitate acute events far faster than traditional metabolic risk factors. Importantly, iatrogenic and conventional risk factors should also be considered when interpreting rapid coronary progression in this single case. Glucocorticoid exposure can exacerbate metabolic and hemodynamic risk profiles (e.g., hyperglycemia, blood pressure elevation, and adverse lipid patterns) and has been associated with dose-dependent increases in cardiovascular events even at low daily doses, which may confound the attribution of lesion progression solely to vasculitis-driven inflammation (10). In addition, systemic glucocorticoids have been linked to a greater risk of venous thromboembolism, and AAV itself is characterized by a heightened thrombotic tendency—particularly during active disease—highlighting a plausible inflammation-coagulation interaction that could contribute to acute atherothrombotic events (11). Moreover, induction regimens that include high-dose glucocorticoids and cyclophosphamide increase susceptibility to serious infections; in the PEXIVAS trial, a reduced-dose glucocorticoid regimen lowered serious infections without compromising efficacy, underscoring the clinical relevance of treatment-related infectious complications (12). Therefore, we also considered whether treatment intensification with rituximab might be warranted in the setting of persistent inflammation. Contemporary recommendations from the European Alliance of Associations for Rheumatology (EULAR) and the American College of Rheumatology/Vasculitis Foundation (ACR/VF) emphasize glucocorticoid tapering and the minimization of treatment-related toxicity during both remission induction and maintenance phases (13). We critically scrutinized the immunosuppressive strategy at the time of the acute coronary syndrome. Although the coronary event represented a localized vascular progression, systemic serological activity was well-controlled (MPO-ANCA had normalized to 8.6 CU from a peak of 61.2 CU), and the patient had received a cyclophosphamide pulse (0.4 g) just weeks prior on May 26, 2025. We decided against further escalating immunosuppression (e.g., adding rituximab or high-dose glucocorticoid pulse) immediately post-PCI for several reasons: 1) Infection Risk: The patient had a significant cumulative cyclophosphamide exposure (~3.0 g) and established pulmonary involvement. Intensifying immunosuppression following an invasive procedure (PCI) would have disproportionately increased the risk of severe opportunistic infections. 2) Recent Treatment: The patient was already under an intensified regimen (recent CYC pulse and maintenance MMF + prednisone 25 mg/day) at the time of the event. 3) Mechanistic Consideration: The acute event was driven by the culmination of anatomical remodeling rather than a *de novo* serological flare. Therefore, we prioritized mechanical revascularization to address the ischemia while maintaining the existing, robust immunosuppressive regimen to stabilize systemic inflammation. Importantly, we critically evaluated the relative contribution of autoimmune-driven inflammation versus traditional cardiovascular risk factors. While the patient’s age and marked hyperglycemia (HbA1c >11%) are established drivers of atherosclerosis, metabolic atherosclerosis typically follows a chronic course characterized by diffuse, heavily calcified plaques. In sharp contrast, our patient exhibited a fulminant progression from non-obstructive disease to critical stenosis within just 16 months. Furthermore, the IVUS phenotype revealed concentric wall thickening with predominantly soft, lipid-rich plaques and minimal calcification. This specific morphological pattern—rapidly evolving soft plaque in the absence of significant calcification—diverges from the classic diabetic phenotype and strongly supports active, vasculitis-associated inflammation as the primary determinant of the coronary severity observed in this case. Furthermore, this distinctive morphology addresses the apparent discrepancy between the non-obstructive findings on the prior CCTA and the subsequent acute event. Unlike the focal, eccentric plaques typical of conventional atherosclerosis, coronary involvement in large-vessel vasculitis (notably Takayasu arteritis) more often presents as diffuse, concentric mural thickening with long-segment, gradually tapering luminal narrowing. On CCTA, the disease may include both stenotic and nonstenotic lesions; therefore, focusing solely on discrete focal stenosis can lead to under-recognition of disease burden (14, 15). Our retrospective identification of diffuse LM tapering supports this interpretation, suggesting that the observed ‘rapid progression’ was essentially the fulminant culmination of this pervasive, unstable inflammatory remodeling process that had been angiographically underestimated.

At the downstream level, the activation of the IL-6-Janus kinase/signal transducer and activator of transcription (JAK–STAT) pathway in vascular smooth muscle cells facilitates a shift from a contractile phenotype toward a synthetic and proinflammatory phenotype, which enhances the expression of matrix metalloproteinases and accelerates extracellular-matrix degradation (16, 17). This remodeling process weakens the fibrous cap and limits mineral deposition, leading to the formation of low-density, lipid-rich, and poorly calcified plaques. This pattern is consistent with the intravascular imaging literature—lesions with a large lipid burden and low calcification signal higher future risk on near-infrared spectroscopy-intravascular ultrasound (NIRS–IVUS)/IVUS natural-history studies (18, 19). In our case, IVUS revealed concentric wall thickening and predominantly soft plaques with minimal calcification, aligning with an inflammation-dominated remodeling phenotype rather than classic heavily calcified atherosclerosis.

Finally, ANCA-associated vasculitis can extend beyond small vessels to involve large arteries; coronary arteritis and aortic complications, including dissection, have been documented (20–22). We also considered other etiologies for large-vessel lesions, such as Takayasu arteritis, IgG4-related aortitis, and heritable connective tissue disorders (e.g., Marfan syndrome). These alternatives were deemed less likely in light of the patient’s overall clinical context and imaging pattern, the absence of supportive systemic features (no claudication or pulse deficits; no typical IgG4-related disease (IgG4-RD) organ involvement or documented IgG4 elevation; no syndromic habitus or family history suggestive of heritable aortopathy), and the presence of MPO-ANCA-positive AAV, although genetic testing and histopathology were not specifically performed. Within this framework, the AAV phenotype provides a coherent explanation for the unusually rapid progression from mild coronary involvement to critical left main and LAD stenosis with occlusion of the circumflex artery, as well as the structural fragility of the large vessel wall culminating in Stanford type A dissection. Overall, the parallel course of persistently elevated inflammatory markers and an IVUS phenotype characterized by concentric wall thickening with predominantly low-calcified, soft plaques is consistent with an inflammation-dominant remodeling process in this setting, underscoring the need for timely immunomodulatory therapy alongside standard antithrombotic and revascularization strategies, in line with contemporary ACR/VF and EULAR recommendations.

## Conclusion

In patients with ANCA-associated vasculitis, persistently active inflammation can drive an inflammation-dominant pattern of coronary remodeling and precipitate acute coronary syndrome, even when lipid levels remain within the normal range over the long term. In clinical practice, inflammatory markers (ANCA, CRP, IL-6, etc.) together with serial coronary imaging should be incorporated into routine evaluation. When appropriate, IVUS or optical coherence tomography (OCT) can be used to further characterize coronary lesions. The presence of large-vessel involvement should also prompt parallel surveillance and individualized intervention, with the aim of improving patient outcomes.

## Data Availability

The original contributions presented in the study are included in the article/**Supplementary Material**. Further inquiries can be directed to the corresponding author.

## Glossary

AAV: ANCA-associated vasculitis
ACR: American College of Rheumatology
ACR/VF: American College of Rheumatology/Vasculitis Foundation
ANCA: antineutrophil cytoplasmic antibody
ApoB: apolipoprotein B
atm: atmosphere (unit of pressure)
BALF: bronchoalveolar lavage fluid
bid: twice daily
BMI: body mass index
BNP: B-type natriuretic peptide
BVAS: Birmingham Vasculitis Activity Score
bpm: beats per minute
CAD: coronary artery disease
CAD-RADS: Coronary Artery Disease–Reporting and Data System
CRP: C-reactive protein
CT: computed tomography
CTA: computed tomography angiography
CTD: connective tissue disease
CU: chemiluminescent units
CYC: cyclophosphamide
DAPT: dual antiplatelet therapy
DES: drug-eluting stent
ECG: electrocardiogram (electrocardiography)
eGFR: estimated glomerular filtration rate
EGPA: eosinophilic granulomatosis with polyangiitis
ENT: ear, nose and throat
ESR: erythrocyte sedimentation rate
EULAR: European Alliance of Associations for Rheumatology
GPA: granulomatosis with polyangiitis
HbA1c: hemoglobin A1c
hs-CRP: high-sensitivity C-reactive protein
hs-cTnT: high-sensitivity cardiac troponin T
IL-6: interleukin-6
ILD: interstitial lung disease
IVUS: intravascular ultrasound
JAK: Janus kinase
LAD: left anterior descending (artery)
LCx: left circumflex (artery)
LDL-C: low-density lipoprotein cholesterol
LM: left main (coronary artery)
LVEF: left ventricular ejection fraction
MMF: mycophenolate mofetil
MPA: microscopic polyangiitis
MPO: myeloperoxidase
MRA: magnetic resonance angiography
MRI: magnetic resonance imaging
NIRS: near-infrared spectroscopy
NSTEMI: non–ST-segment elevation myocardial infarction
NT-proBNP: N-terminal pro–B-type natriuretic peptide
OCT: optical coherence tomography
PCI: percutaneous coronary intervention
PEXIVAS: Plasma Exchange and Glucocorticoids in Severe ANCA-Associated Vasculitis (trial acronym)
PR3: proteinase 3
qd: once daily
RCA: right coronary artery
STAT: signal transducer and activator of transcription
STEMI: ST-segment elevation myocardial infarction
TG: triglycerides
tid: three times daily
TIMI: Thrombolysis in Myocardial Infarction
VTE: venous thromboembolism

## References

[1] Kitching AR Anders HJ Basu N Brouwer E Gordon J Jayne DR . ANCA-associated vasculitis. Nat Rev Dis Primers. (2020) 6:71. doi: 10.1038/s41572-020-0204-y, PMID: 32855422

[2] Zhang C Merdler I Waksman R . Correspondence regarding a published study: Inflammation and cholesterol as predictors of cardiovascular events among patients receiving statin therapy: a collaborative analysis of three randomized trials. Cardiovasc Revasc Med. (2023) 57:106. doi: 10.1016/j.carrev.2023.07.012, PMID: 37558562

[3] Ridker PM Everett BM Thuren T MacFadyen JG Chang WH Ballantyne C . Antiinflammatory therapy with canakinumab for atherosclerotic disease. N Engl J Med. (2017) 377:1119–31. doi: 10.1056/NEJMoa1707914, PMID: 28845751

[4] Suppiah R Robson JC Grayson PC Ponte C Craven A Khalid S . *20*22 American College of Rheumatology/European Alliance of Associations for Rheumatology classification criteria for microscopic polyangiitis. Ann Rheum Dis. (2022) 81:321–6. doi: 10.1136/annrheumdis-2021-221796, PMID: 35110332

[5] Isselbacher EM Preventza O Hamilton Black Iii J Augoustides JG Beck AW Bolen MA . *20*22 ACC/AHA guideline for the diagnosis and management of aortic disease: A report of the american heart association/american college of cardiology joint committee on clinical practice guidelines. J Am Coll Cardiol. (2022) 80:e223–393. doi: 10.1016/j.jacc.2022.08.004, PMID: 36334952 PMC9860464

[6] Kessenbrock K Krumbholz M Schönermarck U Back W Gross WL Werb Z . Netting neutrophils in autoimmune small-vessel vasculitis. Nat Med. (2009) 15:623–5. doi: 10.1038/nm.1959, PMID: 19448636 PMC2760083

[7] Söderberg D Segelmark M . Neutrophil extracellular traps in ANCA-associated vasculitis. Front Immunol. (2016) 7:256. doi: 10.3389/fimmu.2016.00256, PMID: 27446086 PMC4928371

[8] Saffarzadeh M Juenemann C Queisser MA Lochnit G Barreto G Galuska SP . Neutrophil extracellular traps directly induce epithelial and endothelial cell death: a predominant role of histones. PloS One. (2012) 7:e32366. doi: 10.1371/journal.pone.0032366, PMID: 22389696 PMC3289648

[9] Hawkins CL Davies MJ . Role of myeloperoxidase and oxidant formation in the extracellular environment in inflammation-induced tissue damage. Free Radic Biol Med. (2021) 172:633–51. doi: 10.1016/j.freeradbiomed.2021.07.007, PMID: 34246778

[10] Pujades-Rodriguez M Morgan AW Cubbon RM Wu J . Dose-dependent oral glucocorticoid cardiovascular risks in people with immune-mediated inflammatory diseases: A population-based cohort study. PloS Med. (2020) 17:e1003432. doi: 10.1371/journal.pmed.1003432, PMID: 33270649 PMC7714202

[11] Coburn BW Baker JF Hsu JY Wu Q Xie F Curtis JR . Association of cardiovascular outcomes with low-dose glucocorticoid prescription in patients with rheumatoid arthritis. Arthritis Rheumatol. (2024) 76:1585–93. doi: 10.1002/art.42928, PMID: 38923870 PMC11521768

[12] Walsh M Merkel PA Peh CA Szpirt WM Puéchal X Fujimoto S . Plasma exchange and glucocorticoids in severe ANCA-associated vasculitis. N Engl J Med. (2020) 382:622–31. doi: 10.1056/NEJMoa1803537, PMID: 32053298 PMC7325726

[13] Hellmich B Sanchez-Alamo B Schirmer JH Berti A Blockmans D Cid MC . EULAR recommendations for the management of ANCA-associated vasculitis: 2022 update. Ann Rheum Dis. (2024) 83:30–47. doi: 10.1136/ard-2022-223764, PMID: 36927642

[14] Zhu FP Luo S Wang ZJ Jin ZY Zhang LJ Lu GM . Takayasu arteritis: imaging spectrum at multidetector CT angiography. Br J Radiol. (2012) 85:e1282–1292. doi: 10.1259/bjr/25536451, PMID: 23175494 PMC3611735

[15] Soto ME Meléndez-Ramírez G Kimura-Hayama E Meave-Gonzalez A Achenbach S Herrera MC . Coronary CT angiography in Takayasu arteritis. JACC Cardiovasc Imaging. (2011) 4:958–66. doi: 10.1016/j.jcmg.2011.04.019, PMID: 21920333

[16] Watanabe S Mu W Kahn A Jing N Li JH Lan HY . Role of JAK/STAT pathway in IL-6-induced activation of vascular smooth muscle cells. Am J Nephrol. (2004) 24:387–92. doi: 10.1159/000079706, PMID: 15256805

[17] Wang Z Newman WH . Smooth muscle cell migration stimulated by interleukin 6 is associated with cytoskeletal reorganization. J Surg Res. (2003) 111:261–6. doi: 10.1016/S0022-4804(03)00087-8, PMID: 12850472

[18] Erlinge D Maehara A Ben-Yehuda O Bøtker HE Maeng M Kjøller-Hansen L . Identification of vulnerable plaques and patients by intracoronary near-infrared spectroscopy and ultrasound (PROSPECT II): a prospective natural history study. Lancet. (2021) 397:985–95. doi: 10.1016/S0140-6736(21)00249-X, PMID: 33714389

[19] Suh WM Seto AH Margey RJ Cruz-Gonzalez I Jang IK . Intravascular detection of the vulnerable plaque. Circ Cardiovasc Imaging. (2011) 4:169–78. doi: 10.1161/CIRCIMAGING.110.958777, PMID: 21406663

[20] Chirinos JA Tamariz LJ Lopes G Del Carpio F Zhang X Milikowski C . Large vessel involvement in ANCA-associated vasculitides: report of a case and review of the literature. Clin Rheumatol. (2004) 23:152–9. doi: 10.1007/s10067-003-0816-0, PMID: 15045631

[21] Monghal V Puéchal X Smets P Vandergheynst F Michel M Diot E . Large-vessel involvement in ANCA-associated vasculitis: A multicenter case-control study. Semin Arthritis Rheumatol. (2024) 67:152475. doi: 10.1016/j.semarthrit.2024.152475, PMID: 38810568

[22] Pan L Yan JH Gao FQ Li H Han SS Cao GH . Case report of a 28-year-old man with aortic dissection and pulmonary shadow due to granulomatosis with polyangiitis. BMC Pulm Med. (2019) 19:122. doi: 10.1186/s12890-019-0884-9, PMID: 31286925 PMC6615146

