# Supplementary figures and images for: Case Report: Rapid progression of inflammation-driven coronary artery lesions in a normolipidemic patient with ANCA-associated vasculitis complicated by Stanford type A aortic dissection
Source: Front Immunol. 2026 Mar 12;17:1736895. doi: 10.3389/fimmu.2026.1736895 (PMC13017790; doi:10.3389/fimmu.2026.1736895)

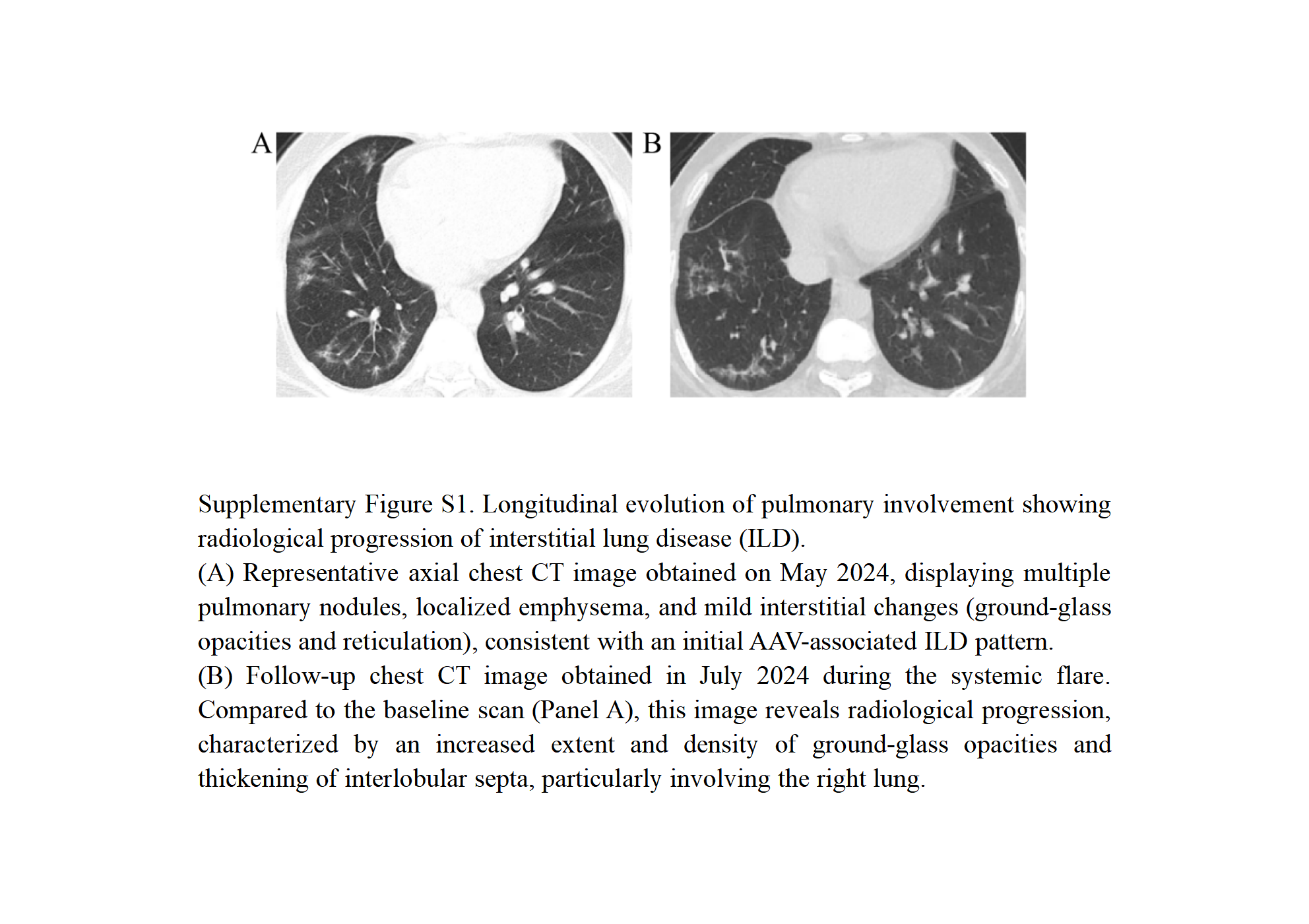

Supplement: Supplementary file 1 [file Image1.tif]
