# Supplementary material for: Case Report: Rapid progression of inflammation-driven coronary artery lesions in a normolipidemic patient with ANCA-associated vasculitis complicated by Stanford type A aortic dissection
Source: Front Immunol. 2026 Mar 12;17:1736895. doi: 10.3389/fimmu.2026.1736895 (PMC13017790; doi:10.3389/fimmu.2026.1736895)
Supplement: Supplementary file 2 [file Table1.docx]

**Supplementary Table S1. Intravenous cyclophosphamide (CYC) pulse regimen between July 2024 and May 2025**

| **Date**  **(YYYY-MM-DD)** | **CYC dose per infusion**  **(g, IV)** | **Cumulative CYC dose**  **(g)** | **Notes** |
| --- | --- | --- | --- |
| 2024-07-12 | 0.2 | 0.2 | Intermittent IV pulse |
| 2024-07-15 | 0.2 | 0.4 | Intermittent IV pulse |
| 2024-08-20 | 0.4 | 0.8 | Intermittent IV pulse |
| 2024-10-05 | 0.4 | 1.2 | Intermittent IV pulse |
| 2024-10-21 | 0.4 | 1.6 | Intermittent IV pulse |
| 2025-01-01 | 0.2 | 1.8 | Intermittent IV pulse |
| 2025-01-21 | 0.2 | 2.0 | Intermittent IV pulse |
| 2025-02-24 | 0.2 | 2.2 | Intermittent IV pulse |
| 2025-04-23 | 0.2 | 2.4 | Intermittent IV pulse |
| 2025-05-26 | 0.4 | 2.8 | Intermittent IV pulse |
