# Supplementary material for: Case Report: Rapid progression of inflammation-driven coronary artery lesions in a normolipidemic patient with ANCA-associated vasculitis complicated by Stanford type A aortic dissection
Source: Front Immunol. 2026 Mar 12;17:1736895. doi: 10.3389/fimmu.2026.1736895 (PMC13017790; doi:10.3389/fimmu.2026.1736895)
Supplement: Supplementary file 3 [file Table2.docx]

**Supplementary Table S2. Baseline conventional cardiovascular risk profiling and glycemic indices (repeated measurements within the preceding year)**

1. **Anthropometrics and blood pressure**

| **Variable** | **Unit** | **Serial measurements**  **(month/year)** | **Summary (range/description)** |
| --- | --- | --- | --- |
| Height | cm | — | 153 |
| Weight | kg | 07/2024–06/2025 | 50–54 |
| BMI (calculated) | kg/m² | 07/2024–06/2025 | 21.36–23.07 (non-obese) |
| Smoking history | — | — | No smoking history (self-reported) |
| Family history of premature CAD | — | — | No family history (self-reported) |
| Blood pressure | mmHg | 07/2024–06/2025 | Repeatedly within normotensive-to-low range; no documented sustained hypertension |

1. **Glycemic indices**

| **Variable** | **Unit** | **Serial measurements**  **(month/year)** | **Summary**  **(range/description)** |
| --- | --- | --- | --- |
| Fasting plasma glucose | mmol/L | 07/2024–06/2025 | 8.31–10.81 |
| Random glucose | mmol/L | 07/2024–06/2025 | Up to 18.48 |
| 2-h postprandial glucose (if available) | mmol/L | 07/2024–06/2025 | 13.0–16.1 (available readings) |
| HbA1c | % | 07/2024–06/2025 | 11.1–11.7 |
