# Supplementary material for: Case Report: Rapid progression of inflammation-driven coronary artery lesions in a normolipidemic patient with ANCA-associated vasculitis complicated by Stanford type A aortic dissection
Source: Front Immunol. 2026 Mar 12;17:1736895. doi: 10.3389/fimmu.2026.1736895 (PMC13017790; doi:10.3389/fimmu.2026.1736895)
Supplement: Supplementary file 4 [file Table3.docx]

**Supplementary Table S3.Serial renal function and urinalysis results (07/2024–06/2025)**

1. **Serum creatinine and eGFR**

| **Date (YYYY-MM-DD)** | **Serum creatinine (μmol/L)** | **eGFR (mL/min/1.73 m²)** |
| --- | --- | --- |
| 2024-07-03 | 58.6 | NA |
| 2024-07-12 | 38.8 | 113 |
| 2024-08-14 | 42.2 | 110 |
| 2024-11-22 | 45.1 | 107.83 |
| 2024-12-17 | 48.1 | 105.57 |
| 2024-12-31 | 47.3 | 106.15 |
| 2025-05-26 | 40.9 | 111.35 |
| 2025-06-24 | 41.3 | 111.00 |

Reference range (lab): creatinine 41–73 μmol/L; eGFR >90.

1. **Urea nitrogen and urinalysis**

| **Date range**  **(YYYY-MM-DD)** | **Urea**  **(mmol/L)** | **Urea/creatinine ratio** | **Urine protein** | **Urinary RBC**  **(sediment)** |
| --- | --- | --- | --- | --- |
| 07/2024–06/2025 (serial) | 4.0–6.2 | 83.16–159.79 | Negative | Negative |

Reference range (lab): urea 2.6–7.5 mmol/L. Urine protein was negative on serial testing, and urinary sediment RBC/HPF was negative.
