# Supplementary material for: Case Report: Rapid progression of inflammation-driven coronary artery lesions in a normolipidemic patient with ANCA-associated vasculitis complicated by Stanford type A aortic dissection
Source: Front Immunol. 2026 Mar 12;17:1736895. doi: 10.3389/fimmu.2026.1736895 (PMC13017790; doi:10.3389/fimmu.2026.1736895)
Supplement: Supplementary file 5 [file Table4.docx]

**Supplementary Table S4. Serial lipid parameters during follow-up (07/2024–06/2025)**

| **Date (YYYY-MM-DD)** | **LDL-C (mmol/L)** | **ApoB (g/L)** | **TG (mmol/L)** |
| --- | --- | --- | --- |
| 2024-07-04 | 2.59 | 0.94 | \| 1.83 \| \| --- \| |
| 2024-11-22 | 2.38 | 0.87 | \| 0.98 \| \| --- \| |
| 2025-06-14 | 2.73 | 0.92 | \| 1.14 \| \| --- \| |

Reference range (lab): LDL-C 0–3.37 mmol/L; ApoB 0.6–1.1 g/L; TG <1.7 mmol/L.
